# Supplementary material for: Global mortality of snakebite envenoming between 1990 and 2019
Source: Nat Commun. 2022 Oct 25;13:6160. doi: 10.1038/s41467-022-33627-9 (PMC9596405; doi:10.1038/s41467-022-33627-9)
Supplement: Supplementary file 1 — Supplementary Information [file 41467_2022_33627_MOESM1_ESM.pdf]

Supplementary Information

Global mortality of snakebite envenoming between 1990 and 2019

Table of Contents

Supplementary Information ***1***

Supplementary Methods ***5***

- **Covariate Selection** **5**
- **Years of Life Lost (YLL) Calculations** **6**
- **Data Sources and Processing** **7**
- **ST-GPR parameters** **13**

Supplementary results ***15***

GATHER checklist ***20***

References ***22***

Supplementary Figures

Supplementary Figure 1: Venomous animal contact CODEm model data. 8
Supplementary Figure 2: Count of unique location-years of data by location used in the snakebite-specific model 9
Supplementary Figure 3: Map of vital registration (VR) and verbal autopsy (VA) data coverage used in the snakebite-specific model. 10
Supplementary Figure 4: Standardised weighted beta coefficients of the covariates used in the ensemble modelling approach 15
Supplementary Figure 5: Age-standardized YLLs due to snakebites in 2019 for both sexes combined 16

Supplementary Tables

Supplementary Table 1. Model covariates in the GBD 2019 and animal-specific ST-GPR models 5
Supplementary Table 2. Residual years of life lost from a death by age 6
Supplementary Table 3: Venomous species-specific ICD codes 7
Supplementary Table 4: Locations where there are no endemic venomous snakes 11
Supplementary Table 5: Venomous snakebite deaths and YLLs in India and its states in 2019 16
Supplementary Table 6: Regional forecast mortality forecasted to 2030, 2040, and 2050. 18
Supplementary Table 7: Guidelines for Accurate and Transparent Health Estimates Reporting (GATHER) checklist 20

Supplementary Methods

Covariate Selection

Supplementary Table 1. Model covariates in the GBD 2019 and animal-specific ST-GPR models

Covariate	GBD 2019 direction	Species-specific direction
Rainfall population-weighted (mm/yr)	1	0
Urbanicity	-1	-1
Proportion of population involved in agricultural activities	1	1
Population-weighted mean temperature	1	0
Absolute value of average latitude	-1	0
Elevation over 1500m (proportion)	-1	0
Elevation under 100m (proportion)	-1	0
Education (years per capita)	-1	-1
Population density (over 1000 ppl/sqkm, proportion)	-1	-1
Population density (under 150 ppl/sqkm, proportion)	1	1
Healthcare Access and Quality Index	-1	-1
Socio-demographic Index	-1	-1
LDI (I$ per capita)	-1	-1
Log-transformed age-standardized SEV scalar: venom	1	1
Proportion of population vulnerable to venomous snakebites[a]	1	1
Mean number of venomous snake species[a]	1	1
a: These covariates were only used in the mortality from venomous snakes model, not the other four species. They were extracted from data used by Longbottom et al.[1] LDI=lag-distributed income. SEV=summary exposure value[2]. ST-GPR=spatiotemporal Gaussian process regression.		

Years of Life Lost (YLL) Calculations

Supplementary Table 2. Residual years of life lost from a death by age

Age	Residual life expectancy (years)
0	87.9
1	87.0
5	83.0
10	78.1
15	73.1
20	68.1
25	63.2
30	58.2
35	53.3
40	48.4
45	43.5
50	38.7
55	34.0
60	29.3
65	24.7
70	20.3
75	16.1
80	12.2
85	8.8
90	6.1
95	3.9
100	2.2
105	1.6
110	1.4

Data Sources and Processing

Supplementary Table 3: Venomous species-specific ICD codes

**Venomous animal**	**ICD-9**	**ICD-10**
Snake	E905.0x	X20.x
Spider	E905.1x	X21.x
Scorpion	E905.2x	X22.x
Bees	E905.3x	X23.x
Other	E905.4x; E905.5x; E905.6x; E905.7x; E905.8x	X24.x; X25.x; X26.x; X27.x; X28.x
Unspecified venomous animal (Considered as garbage codes for redistribution)	E905; E905.9x;	X29.x
Trailing x's represent any value that trails the non-x characters, because every ICD code within the ICD categories was used		

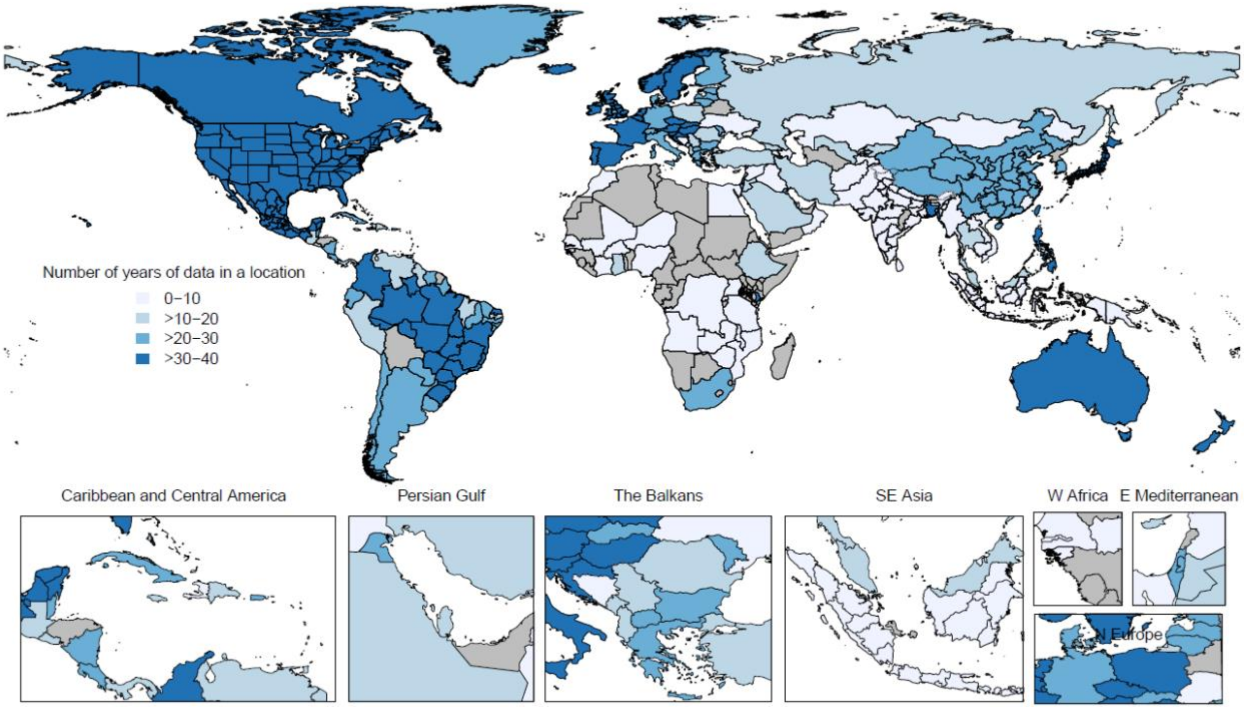

Supplementary Figure 1: Venomous animal contact CODEm model data. Gray represents countries with no data.

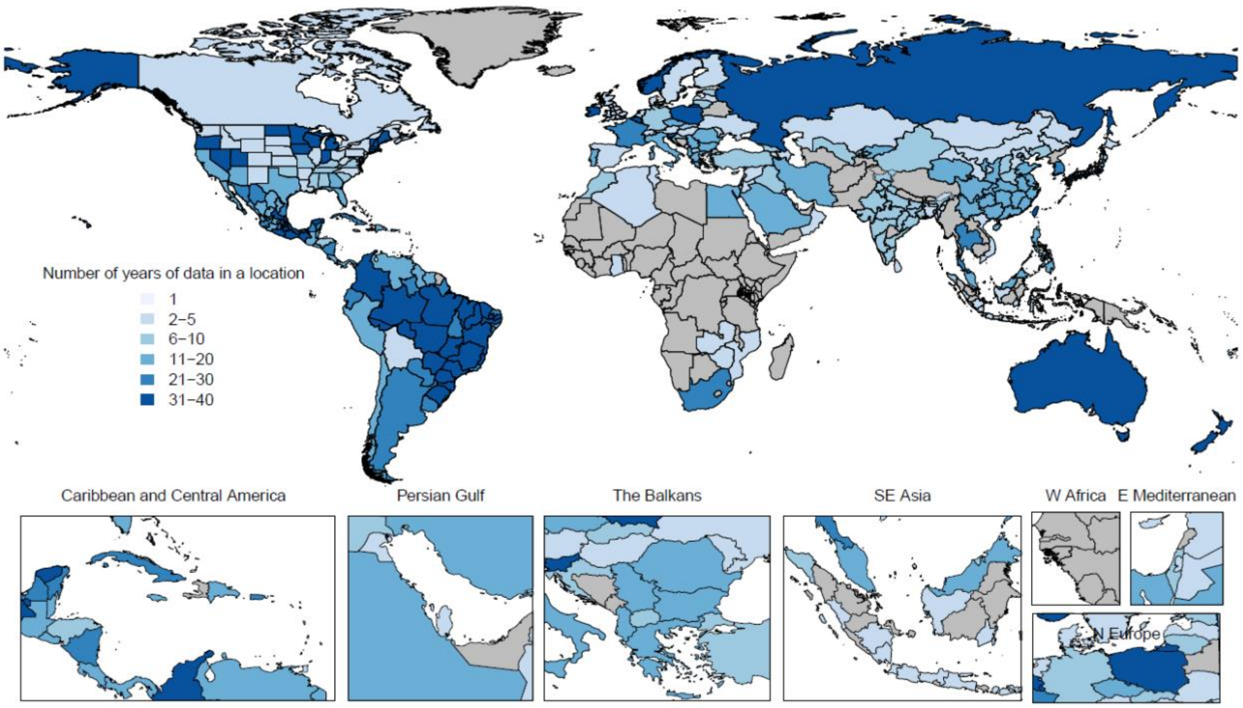

Supplementary Figure 2: Count of unique location-years of data by location used in the snakebite-specific model.

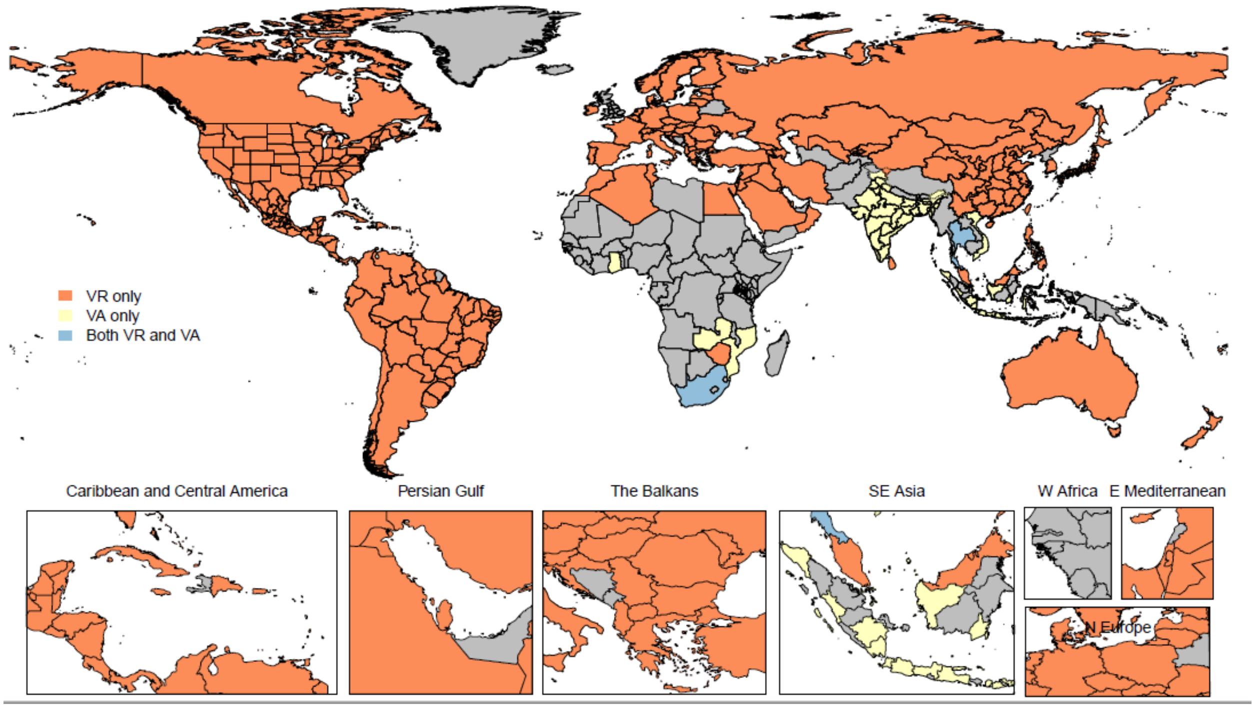

Supplementary Figure 3: Map of vital registration (VR) and verbal autopsy (VA) data coverage used in the snakebite-specific model.

Locations in gray do not have a single source of data.

Supplementary Table 4: Locations where there are no endemic venomous snakes

American Samoa
Antigua and Barbuda
Barbados
Bermuda
Cape Verde
Chile
Comoros
Cook Islands
Cuba
Dominica
Dominican Republic
Federated States of Micronesia
Fiji
Greenland
Grenada
Guam
Haiti
Iceland
Ireland
Jamaica
Kiribati
Madagascar
Maldives
Malta
Marshall Islands
Mauritius
Monaco

Nauru
New Zealand
Niue
Northern Mariana Islands
Palau
Puerto Rico
Saint Kitts and Nevis
Saint Vincent and the Grenadines
Samoa
Seychelles
Solomon Islands
The Bahamas
Tokelau
Tonga
Tuvalu
Vanuatu
Virgin Islands

ST-GPR parameters

Spatiotemporal Gaussian process regression (ST-GPR) has three different hyperparameters that are set to control the amount of temporal, age, and spatial smoothing. Time smoothing follows Equation 1, where j is the observed data point and I is the country-year-age-sex to be predicted. We set λ to be equal to 0.1, causing a high amount of smoothing over time due to a prior expectation that the burden of snakebite would not have significant change year to year.

$$Eq. 1:\ w_t = e^{-\lambda|time_i - time_j|}$$

Age weighting follows equation 2, where j is an observed data point, I is a country-year-age-sex point to be predicted, and ω is the set hyperparameter. We set omega to be 0.5, establishing a medium rate of smoothing over age to allow some effect while also giving ST-GPR the flexibility to follow data points closely.

$$Eq. 2: age\ weight_{i,j} = 0$$

Space weighting follows Eq. 3 below. We set zeta equal to 0.01, creating very little smoothing between countries and subnational locations. We believed there would be significant variation between countries due to ecology, health system strength, and other characteristics, and we allowed ST-GPR to follow the trends in a given location when provided with data to do so.

$$\textit{Eq. 3: Space weight} = \begin{cases} \zeta^0 = 1 & for\ residuals\ within\ country\ i \\ \zeta^1 & for\ residuals\ within\ region\ j\ but\ not\ country\ i \\ \zeta^2 & for\ residuals\ not\ in\ region\ j \end{cases}$$

ST-GPR Covariate Selection

The covariates and their resulting coefficient of the ensemble model are shown below. Each sex is modeled independently. Standardized coefficients allow easy comparisons between covariates on very different scales. The mixed-effects model with nested random effects using GBD regions and data input locations is described below:

$$Snakebite\ mortality\ rate\ per\ 100{,}000 \sim covariates + (1|GBD\ region/location)$$

Due to the varying scales of the different covariates, the strength of their association was standardized. Standardized coefficients are equal to the beta coefficient per unit of the variable times the standard deviation of the covariates over the standard deviation of the input data:

$$\beta_{standardised} = \frac{\beta_{untransformed} * \sigma_{covariate}}{\sigma_{snakebite\ mortality\ rate\ per\ 100{,}000}}$$

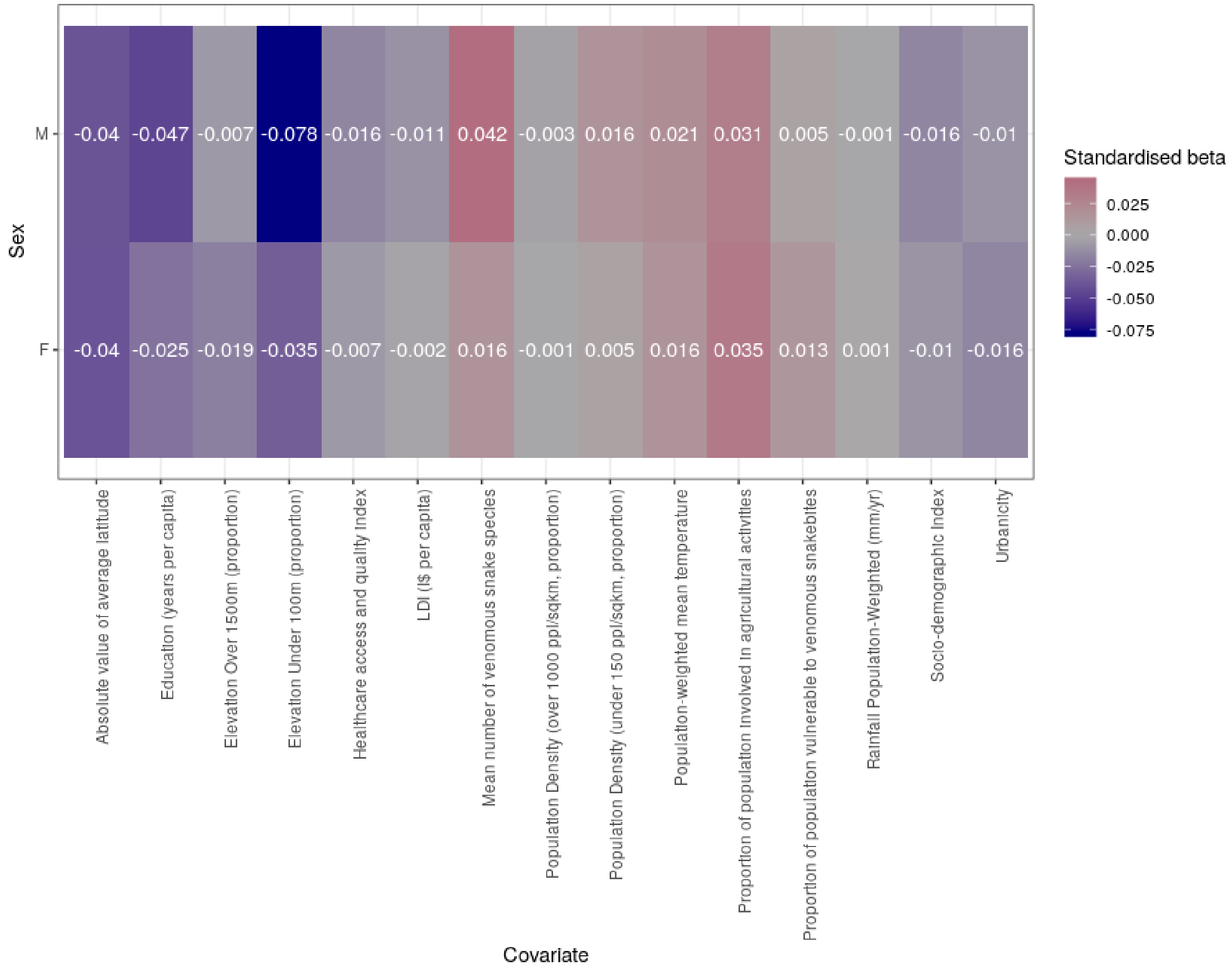

Supplementary Figure 4: Standardised weighted beta coefficients of the covariates used in the ensemble modelling approach

Supplementary results

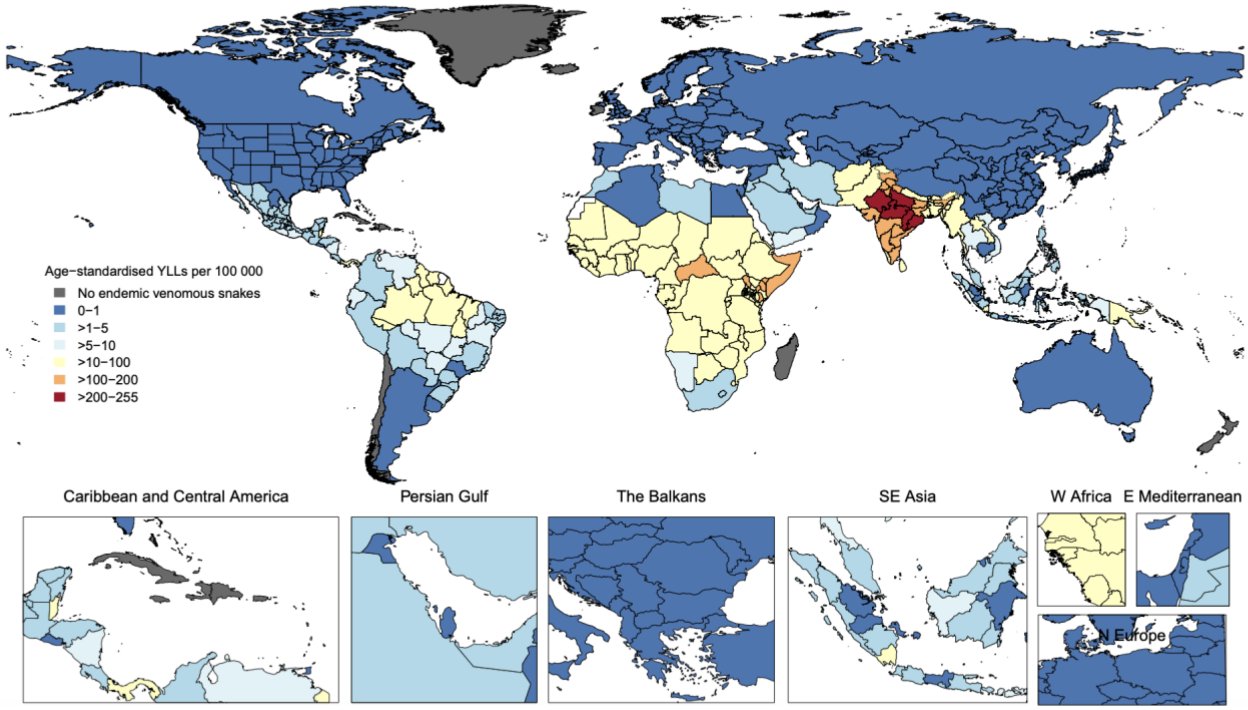

Supplementary Figure 5: Age-standardized YLLs due to snakebites in 2019 for both sexes combined.

GBD 2019 did not publish state-level estimates for China, and each state is colored the estimate of the rate of China's national estimate. Endemic habitat of venomous snakes of medical importance was looked up from the World Health Organization Snakebite Information and Data Platform: https://www.who.int/teams/control-of-neglected-tropical-diseases/snakebite-envenoming/snakebite-information-and-data-platform/overview#tab=tab_1.

Supplementary Table 5: Venomous snakebite deaths and YLLs in India and its states in 2019.

Location	Deaths - Count, 2019	Deaths - Age-standardized rate, 2019	YLLs - Count, 2019	YLLs - Age-standardized rate, 2019
Andhra Pradesh	2,310 (1,190 to 3,110)	4.19 (2.15 to 5.63)	89,300 (47,800 to 122,000)	163 (87 to 223)
Arunachal Pradesh	21 (15 to 42)	1.68 (1.22 to 3.27)	1,030 (718 to 2,100)	66 (46 to 131)
Assam	768 (605 to 1,000)	2.44 (1.92 to 3.14)	37,500 (29,200 to 49,900)	107 (84 to 141)
Bihar	3,760 (1,570 to 5,290)	3.63 (1.53 to 5.05)	190,000 (76,500 to 276,000)	160 (65 to 229)
Chhattisgarh	1,810 (962 to 2,370)	6.50 (3.52 to 8.42)	80,700 (44,300 to 108,000)	263 (143 to 349)
Delhi	211 (148 to 275)	1.22 (0.86 to 1.58)	9,530 (6,860 to 12,600)	50 (36 to 67)
Goa	19 (14 to 25)	1.11 (0.81 to 1.48)	673 (487 to 910)	41 (30 to 55)
Gujarat	2,730 (1,490 to 3,440)	4.23 (2.29 to 5.34)	124,000 (69,000 to 158,000)	178 (99 to 228)
Haryana	710 (535 to 891)	2.62 (1.99 to 3.30)	33,300 (25,500 to 42,400)	116 (88 to 148)
Himachal Pradesh	268 (157 to 339)	3.52 (2.04 to 4.44)	10,600 (6,170 to 13,500)	138 (81 to 175)
Jammu & Kashmir and Ladakh	478 (172 to 623)	3.97 (1.41 to 5.15)	21,800 (8,000 to 28,700)	163 (59 to 214)
Jharkhand	1,040 (580 to 1,480)	3.20 (1.78 to 4.44)	46,600 (26,200 to 67,700)	129 (73 to 186)
Karnataka	2,100 (1,590 to 2,600)	3.19 (2.40 to 3.94)	92,800 (70,800 to 115,000)	136 (104 to 168)
Kerala	307 (238 to 453)	0.74 (0.58 to 1.13)	10,700 (8,230 to 17,100)	28 (21 to 45)
Madhya Pradesh	4,390 (2,470 to 5,790)	5.68 (3.08 to 7.39)	215,000 (125,000 to 289,000)	249 (141 to 332)
Maharashtra	4,010 (2,390 to 5,070)	3.25 (1.95 to 4.08)	172,000 (106,000 to 214,000)	136 (85 to 170)
Manipur	35 (25 to 59)	1.19 (0.85 to 2.00)	1,560 (1,090 to 2,620)	47 (33 to 78)
Meghalaya	33 (22 to 63)	1.29 (0.92 to 2.50)	1,580 (1,070 to 3,080)	51 (35 to 97)

Mizoram	11 (6 to 29)	1.05 (0.63 to 2.86)	490 (293 to 1,290)	41 (25 to 107)
Nagaland	22 (17 to 31)	1.49 (1.12 to 2.06)	1,060 (769 to 1,530)	59 (44 to 83)
Odisha	2,250 (1,360 to 3,030)	5.00 (3.01 to 6.69)	103,000 (61,800 to 141,000)	222 (132 to 306)
Other Union Territories	33 (22 to 49)	0.94 (0.65 to 1.38)	1,310 (882 to 2,000)	35 (24 to 53)
Punjab	881 (633 to 1,110)	2.78 (2.00 to 3.51)	37,100 (26,900 to 47,000)	117 (85 to 146)
Rajasthan	4,070 (2,440 to 5,230)	5.80 (3.48 to 7.44)	205,000 (122,000 to 265,000)	261 (151 to 336)
Sikkim	7 (4 to 10)	1.18 (0.77 to 1.71)	270 (175 to 411)	44 (29 to 65)
Tamil Nadu	2,780 (1,720 to 3,530)	3.40 (2.13 to 4.30)	111,000 (74,700 to 141,000)	135 (91 to 171)
Telangana	1,550 (780 to 2,180)	4.38 (2.19 to 6.05)	64,900 (34,200 to 91,100)	171 (89 to 239)
Tripura	26 (17 to 68)	0.69 (0.46 to 1.77)	1,080 (700 to 2,860)	27 (18 to 70)
Uttar Pradesh	12,000 (5,230 to 16,100)	6.02 (2.60 to 7.99)	566,000 (252,000 to 784,000)	247 (109 to 337)
Uttarakhand	423 (231 to 591)	3.91 (2.16 to 5.46)	17,800 (10,200 to 24,700)	153 (86 to 213)
West Bengal	2,090 (1,220 to 2,730)	2.16 (1.23 to 2.81)	91,600 (54,200 to 120,000)	92 (54 to 122)
**India**	**51,100 (29,600 to 64,100)**	**4.00 (2.31 to 5.01)**	**2,340,000 (1,350,000 to 2,970,000)**	**171 (99 to 218)**

Supplementary Table 6: Regional forecast mortality forecasted to 2030, 2040, and 2050.

Region	Deaths (Absolute number)					Age-standardized mortality rate per 100,000				
	2019*	2020	2030	2040	2050	2019*	2020	2030	2040	2050
Andean Latin America	47 (16 to 60)	68 (15 to 83)	86 (12 to 112)	106 (10 to 149)	129 (7 to 199)	0.08 (0.03 to 0.10)	0.11 (0.02 to 0.13)	0.11 (0.02 to 0.15)	0.12 (0.01 to 0.16)	0.12 (0.01 to 0.18)
Australasia	1 (1 to 2)	1 (1 to 1)	1 (1 to 1)	1 (1 to 1)	1 (0 to 1)	0.00 (0.00 to 0.00)	0.00 (0.00 to 0.00)	0.00 (0.00 to 0.00)	0.00 (0.00 to 0.00)	0.00 (0.00 to 0.00)
Caribbean	12 (9 to 15)	18 (14 to 21)	35 (24 to 44)	70 (42 to 94)	149 (74 to 216)	0.02 (0.02 to 0.03)	0.04 (0.03 to 0.04)	0.06 (0.04 to 0.07)	0.10 (0.06 to 0.14)	0.18 (0.09 to 0.26)
Central Asia	9 (8 to 10)	10 (9 to 11)	9 (8 to 11)	8 (7 to 11)	8 (6 to 10)	0.01 (0.01 to 0.01)	0.01 (0.01 to 0.01)	0.01 (0.01 to 0.01)	0.01 (0.01 to 0.01)	0.01 (0.00 to 0.01)
Central Europe	5 (4 to 6)	6 (5 to 6)	5 (5 to 6)	5 (4 to 5)	4 (3 to 5)	0.00 (0.00 to 0.00)	0.00 (0.00 to 0.00)	0.00 (0.00 to 0.00)	0.00 (0.00 to 0.00)	0.00 (0.00 to 0.00)
Central Latin America	210 (174 to 255)	189 (170 to 206)	147 (127 to 168)	112 (92 to 133)	83 (65 to 102)	0.09 (0.07 to 0.11)	0.07 (0.07 to 0.08)	0.05 (0.04 to 0.05)	0.03 (0.03 to 0.04)	0.02 (0.02 to 0.02)
Central Sub-Saharan Africa	791 (507 to 1,355)	883 (564 to 1,579)	1,164 (656 to 2,416)	1,535 (752 to 3,840)	2,039 (853 to 6,028)	1.25 (0.83 to 1.82)	1.26 (0.84 to 2.05)	1.18 (0.68 to 2.40)	1.13 (0.57 to 2.71)	1.09 (0.47 to 3.09)
East Asia	230 (176 to 280)	145 (128 to 191)	85 (69 to 159)	48 (35 to 124)	26 (17 to 90)	0.01 (0.01 to 0.01)	0.01 (0.01 to 0.01)	0.00 (0.00 to 0.01)	0.00 (0.00 to 0.00)	0.00 (0.00 to 0.00)
Eastern Europe	22 (19 to 26)	19 (18 to 21)	13 (12 to 15)	9 (8 to 10)	6 (5 to 7)	0.01 (0.01 to 0.01)	0.01 (0.01 to 0.01)	0.01 (0.00 to 0.01)	0.00 (0.00 to 0.00)	0.00 (0.00 to 0.00)
Eastern Sub-Saharan Africa	2,102 (1,573 to 3,002)	2,208 (1,634 to 3,116)	2,591 (1,819 to 3,955)	3,109 (2,051 to 5,205)	3,738 (2,268 to 6,771)	1.19 (0.83 to 1.61)	1.10 (0.79 to 1.54)	0.90 (0.62 to 1.36)	0.74 (0.48 to 1.22)	0.61 (0.37 to 1.09)
High-income Asia Pacific	9 (7 to 11)	8 (6 to 9)	6 (4 to 7)	4 (3 to 5)	3 (2 to 4)	0.00 (0.00 to 0.00)	0.00 (0.00 to 0.00)	0.00 (0.00 to 0.00)	0.00 (0.00 to 0.00)	0.00 (0.00 to 0.00)
High-income North America	17 (16 to 19)	16 (14 to 17)	16 (14 to 17)	15 (14 to 17)	15 (13 to 17)	0.00 (0.00 to 0.00)	0.00 (0.00 to 0.00)	0.00 (0.00 to 0.00)	0.00 (0.00 to 0.00)	0.00 (0.00 to 0.00)

North Africa and Middle East	350 (243 to 485)	379 (272 to 493)	326 (226 to 478)	283 (180 to 468)	246 (140 to 460)	0.06 (0.05 to 0.09)	0.06 (0.05 to 0.08)	0.05 (0.03 to 0.07)	0.04 (0.02 to 0.06)	0.03 (0.02 to 0.05)
Oceania	69 (40 to 108)	77 (42 to 116)	90 (47 to 140)	106 (54 to 171)	125 (59 to 217)	0.65 (0.38 to 1.03)	0.64 (0.36 to 1.00)	0.63 (0.34 to 0.99)	0.62 (0.31 to 1.00)	0.61 (0.29 to 1.06)
South Asia	54,588 (31,838 to 68,321)	60,538 (34,100 to 75,645)	58,433 (32,577 to 85,624)	56,447 (30,908 to 95,744)	54,241 (28,352 to 106,833)	3.37 (1.96 to 4.19)	3.64 (2.06 to 4.56)	3.03 (1.70 to 4.45)	2.55 (1.41 to 4.33)	2.17 (1.14 to 4.27)
Southeast Asia	801 (581 to 961)	840 (603 to 968)	689 (500 to 870)	557 (395 to 791)	438 (287 to 693)	0.14 (0.10 to 0.16)	0.14 (0.10 to 0.16)	0.09 (0.07 to 0.12)	0.06 (0.04 to 0.09)	0.04 (0.03 to 0.07)
Southern Latin America	3 (3 to 4)	5 (4 to 5)	8 (6 to 9)	12 (10 to 15)	18 (14 to 24)	0.00 (0.00 to 0.01)	0.01 (0.01 to 0.01)	0.01 (0.01 to 0.01)	0.01 (0.01 to 0.02)	0.02 (0.01 to 0.02)
Southern Sub-Saharan Africa	71 (56 to 90)	78 (62 to 98)	90 (68 to 122)	107 (76 to 152)	127 (84 to 200)	0.12 (0.09 to 0.15)	0.12 (0.10 to 0.15)	0.11 (0.09 to 0.15)	0.11 (0.08 to 0.15)	0.10 (0.07 to 0.16)
Tropical Latin America	240 (224 to 261)	204 (188 to 220)	185 (165 to 208)	164 (141 to 192)	140 (115 to 171)	0.11 (0.10 to 0.12)	0.09 (0.08 to 0.09)	0.06 (0.06 to 0.07)	0.05 (0.04 to 0.06)	0.04 (0.03 to 0.04)
Western Europe	14 (12 to 15)	14 (13 to 15)	14 (13 to 15)	14 (12 to 16)	14 (12 to 15)	0.00 (0.00 to 0.00)	0.00 (0.00 to 0.00)	0.00 (0.00 to 0.00)	0.00 (0.00 to 0.00)	0.00 (0.00 to 0.00)
Western Sub-Saharan Africa	3,822 (2,682 to 6,000)	3,903 (2,756 to 5,900)	4,808 (3,093 to 8,106)	5,922 (3,548 to 10,915)	7,222 (3,935 to 14,706)	1.42 (1.03 to 2.06)	1.42 (1.01 to 2.10)	1.24 (0.81 to 2.03)	1.09 (0.65 to 1.97)	0.97 (0.53 to 1.95)
**Global**	**63,415 (38,930 to 78,633)**	**69,608 (42,094 to 86,040)**	**68,799 (41,372 to 97,613)**	**68,633 (40,347 to 111,231)**	**68,771 (39,108 to 125,599)**	**0.81 (0.50 to 1.00)**	**0.87 (0.53 to 1.08)**	**0.75 (0.45 to 1.07)**	**0.66 (0.39 to 1.08)**	**0.59 (0.34 to 1.08)**

*: 2019 data are our snakebite mortality estimates, while 2020, 2030, 2040, and 2050 estimates are the results of the forecasting model.

GATHER checklist

Supplementary Table 7: Guidelines for Accurate and Transparent Health Estimates Reporting (GATHER) checklist

Item #	Checklist item	Reported on page #
Objectives and funding		
1	Define the indicator(s), populations (including age, sex, and geographic entities), and time period(s) for which estimates were made.	Main text (Methods)
2	List the funding sources for the work.	Abstract (Funding), Acknowledgments
Data Inputs		
For all data inputs from multiple sources that are synthesized as part of the study:		
3	Describe how the data were identified and how the data were accessed.	Main manuscript methods and appendix
4	Specify the inclusion and exclusion criteria. Identify all ad-hoc exclusions.	Main manuscript methods and appendix
5	Provide information on all included data sources and their main characteristics. For each data source used, report reference information or contact name/institution, population represented, data collection method, year(s) of data collection, sex and age range, diagnostic criteria or measurement method, and sample size, as relevant.	Main manuscript methods and appendix Sections 3-4. Detailed data sources for each component available online at GBD Input Data Sources Tool (http://ghdx.healthdata.org/gbd-2019/data-input-sources)
6	Identify and describe any categories of input data that have potentially important biases (e.g., based on characteristics listed in item 5).	Main manuscript methods and appendix
For data inputs that contribute to the analysis but were not synthesized as part of the study:		
7	Describe and give sources for any other data inputs.	Main manuscript methods and appendix
For all data inputs:		
8	Provide all data inputs in a file format from which data can be efficiently extracted (e.g., a spreadsheet rather than a PDF), including all relevant meta-data listed in item 5. For any data inputs that cannot be shared because of ethical or legal reasons, such as	Detailed data sources for each component available online (https://ghdx.healthdata.org/gbd-2019)

	third-party ownership, provide a contact name or the name of the institution that retains the right to the data.	
Data analysis		
9	Provide a conceptual overview of the data analysis method. A diagram may be helpful.	Manuscript methods and appendix
10	Provide a detailed description of all steps of the analysis, including mathematical formulae. This description should cover, as relevant, data cleaning, data pre-processing, data adjustments and weighting of data sources, and mathematical or statistical model(s).	Manuscript methods and appendix
11	Describe how candidate models were evaluated and how the final model(s) were selected.	Manuscript methods and appendix
12	Provide the results of an evaluation of model performance, if done, as well as the results of any relevant sensitivity analysis.	
13	Describe methods for calculating uncertainty of the estimates. State which sources of uncertainty were, and were not, accounted for in the uncertainty analysis.	Manuscript methods and appendix
14	State how analytic or statistical source code used to generate estimates can be accessed.	Statistical code used in GBD is published on GitHub, including core modelling code for ST-GPR and CODEm
Results and Discussion		
15	Provide published estimates in a file format from which data can be efficiently extracted.	Main text, and GBD 2019 venomous animal contact are publicly available from the GHDx online results tool (http://ghdx.healthdata.org/gbd-results-tool)
16	Report a quantitative measure of the uncertainty of the estimates (e.g. uncertainty intervals).	Main text estimates include 95% uncertainty intervals
17	Interpret results in light of existing evidence. If updating a previous set of estimates, describe the reasons for changes in estimates.	Main text discussion
18	Discuss limitations of the estimates. Include a discussion of any modelling assumptions or data limitations that affect interpretation of the estimates.	Main text discussion

References

1. Longbottom, J. *et al.* Vulnerability to snakebite envenoming: a global mapping of hotspots. *The Lancet* **392**, 673–684 (2018).
2. Stanaway, J. D. *et al.* Global, regional, and national comparative risk assessment of 84 behavioural, environmental and occupational, and metabolic risks or clusters of risks for 195 countries and territories, 1990–2017: a systematic analysis for the Global Burden of Disease Study 2017. *The Lancet* **392**, 1923–1994 (2018).
