# Supplementary material for: Global mortality of snakebite envenoming between 1990 and 2019
Source: Nat Commun. 2022 Oct 25;13:6160. doi: 10.1038/s41467-022-33627-9 (PMC9596405; doi:10.1038/s41467-022-33627-9)
Supplement: Supplementary file 3 — Description of Additional Supplementary Files [file 41467_2022_33627_MOESM3_ESM.pdf]

Title: Supplementary Data 1:
Description: Snakebite envenoming results of the count, age-standardized rate, and percent change 1990-2019 of deaths and years of life lost (YLLs). Green rows represent the global or regional count or rate. The rows below each green region are the countries that make up the region. Endemic venomous snake is based off the WHO Snakebite Information and Data Platform.[31]

Title: Supplementary Data 2:
Description: Input data source citations for GBD 2019 venomous animal contact model.
