# Supplementary material for: Global mortality of snakebite envenoming between 1990 and 2019
Source: Nat Commun. 2022 Oct 25;13:6160. doi: 10.1038/s41467-022-33627-9 (PMC9596405; doi:10.1038/s41467-022-33627-9)
Supplement: Supplementary file 6 — Reporting Summary [file 41467_2022_33627_MOESM6_ESM.pdf]

nature portfolio

Corresponding author(s): Nicholas Roberts

Last updated by author(s): Jul 12, 2022

Reporting Summary

Nature Portfolio wishes to improve the reproducibility of the work that we publish. This form provides structure for consistency and transparency in reporting. For further information on Nature Portfolio policies, see our Editorial Policies and the Editorial Policy Checklist.

Statistics

For all statistical analyses, confirm that the following items are present in the figure legend, table legend, main text, or Methods section.

n/a	Confirmed	
☐	☒	The exact sample size (n) for each experimental group/condition, given as a discrete number and unit of measurement
☐	☒	A statement on whether measurements were taken from distinct samples or whether the same sample was measured repeatedly
☒	☐	The statistical test(s) used AND whether they are one- or two-sided<br>*Only common tests should be described solely by name; describe more complex techniques in the Methods section.*
☐	☒	A description of all covariates tested
☐	☒	A description of any assumptions or corrections, such as tests of normality and adjustment for multiple comparisons
☐	☒	A full description of the statistical parameters including central tendency (e.g. means) or other basic estimates (e.g. regression coefficient) AND variation (e.g. standard deviation) or associated estimates of uncertainty (e.g. confidence intervals)
☒	☐	For null hypothesis testing, the test statistic (e.g. F, t, r) with confidence intervals, effect sizes, degrees of freedom and P value noted<br>*Give P values as exact values whenever suitable.*
☐	☒	For Bayesian analysis, information on the choice of priors and Markov chain Monte Carlo settings
☒	☐	For hierarchical and complex designs, identification of the appropriate level for tests and full reporting of outcomes
☒	☐	Estimates of effect sizes (e.g. Cohen's d, Pearson's r), indicating how they were calculated

Our web collection on statistics for biologists contains articles on many of the points above.

Software and code

Policy information about availability of computer code

Data collection	No primary data collection was carried out for these analyses.
Data analysis	All code used for the GBD 2019 analyses is publicly available online at https://ghdx.healthdata.org/gbd-2019/code and custom code for the snakebite envenomation analysis is publicly available online at https://github.com/nlr4002/Snakebite_Envenomation.

For manuscripts utilizing custom algorithms or software that are central to the research but not yet described in published literature, software must be made available to editors and reviewers. We strongly encourage code deposition in a community repository (e.g. GitHub). See the Nature Portfolio guidelines for submitting code & software for further information.

Data

Policy information about availability of data

All manuscripts must include a data availability statement. This statement should provide the following information, where applicable:

- Accession codes, unique identifiers, or web links for publicly available datasets
- A description of any restrictions on data availability
- For clinical datasets or third party data, please ensure that the statement adheres to our policy

The findings of this study are supported by data that are available in public online repositories, data that are publicly available on request from the data provider, and data that are not publicly available due to restrictions by the data provider and which were used under license for the current study. Details on data sources can be found on the GHDx website including information about the data provider and links to where the data can be accessed or requested (where available) at https://ghdx.healthdata.org/gbd-2019/data-input-sources?components=4&causes=710&locations=1. We have also provided maps of the data included in our models in Supplementary Figures 1-3. Source data are provided with this paper for Figures 1-5.
Information on whether or not venomous snakes inhabited a country was extracted from the World Health Organization Venomous Snakes Distribution and Species

March 2021

Risk Categories database, which is available here: https://apps.who.int/bloodproducts/snakeantivenoms/database/.

Field-specific reporting

Please select the one below that is the best fit for your research. If you are not sure, read the appropriate sections before making your selection.

- [x] Life sciences
- [ ] Behavioural & social sciences
- [ ] Ecological, evolutionary & environmental sciences

For a reference copy of the document with all sections, see nature.com/documents/nr-reporting-summary-flat.pdf

Life sciences study design

All studies must disclose on these points even when the disclosure is negative.

Sample size	Sample size was defined as the population of every location used in our analysis. State-, country-, region-, super-region, and global-level populations are estimated as part of the Global Burden of Disease Study 2019, and methods are described here https://www.thelancet.com/article/S0140-6736(20)30677-2/fulltext
Data exclusions	Select data sources were excluded for the following reasons: missing survey weights, missing sex or age variable, or untrustworthy data (as determined by the survey administrator or by inspection). Data availability plots for snakebite envenoming mortality are available in Supplementary Figures 1-3.
Replication	This is an observational study using many years of verbal autopsy survey and official government vital statistics data and in principle could be replicated. Due to the time required to extract and process all data, as well as to run the statistical models, we have not undertaken an explicit replication analysis.
Randomization	Randomization was not relevant to this study. This analysis is an observational study and there were no experimental groups.
Blinding	Blinding was not relevant to this study. This was an observational study using survey and report data.

Reporting for specific materials, systems and methods

We require information from authors about some types of materials, experimental systems and methods used in many studies. Here, indicate whether each material, system or method listed is relevant to your study. If you are not sure if a list item applies to your research, read the appropriate section before selecting a response.

Materials & experimental systems

n/a	Involved in the study
x	Antibodies
x	Eukaryotic cell lines
x	Palaeontology and archaeology
x	Animals and other organisms
x	Human research participants
x	Clinical data
x	Dual use research of concern

Methods

n/a	Involved in the study
x	ChIP-seq
x	Flow cytometry
x	MRI-based neuroimaging
